# The immune modulatory effects of umbilical cord-derived mesenchymal stromal cells in severe COVID-19 pneumonia

**DOI:** 10.1186/s13287-021-02376-9

**Published:** 2021-06-02

**Authors:** Rachele Ciccocioppo, Davide Gibellini, Giuseppe Astori, Martina Bernardi, Angela Bozza, Katia Chieregato, Francesca Elice, Stefano Ugel, Simone Caligola, Francesco De Sanctis, Stefania Canè, Alessandra Fiore, Rosalinda Trovato, Antonio Vella, Varvara Petrova, Giuseppe Amodeo, Monica Santimaria, Annarita Mazzariol, Luca Frulloni, Marco Ruggeri, Enrico Polati, Vincenzo Bronte

**Affiliations:** 1 Gastroenterology Unit, Department of Medicine, A.O.U.I. Policlinico G.B. Rossi & University of Verona, Piazzale L.A. Scuro, 10, 37134 Verona, Italy; 2 Microbiology Section, Department of Diagnostics and Public Health, University of Verona, Verona, Italy; 3 Laboratory of Advanced Cellular Therapies, Hematology Unit, San Bortolo Hospital, A.U.L.S.S. 8 “Berica”, Vicenza, Italy; 4 Hematology Unit, San Bortolo Hospital, A.U.L.S.S. 8 “Berica”, Vicenza, Italy; 5 Immunology Unit, Department of Medicine, A.O.U.I. Policlinico G.B. Rossi & University of Verona, Verona, Italy; 6 Nuclear Medicine Unit, San Bortolo Hospital, A.U.L.S.S. 8 “Berica”, Vicenza, Italy; 7 Intensive Care Unit, Department of Surgery, Dentistry, Maternity and Infant, A.O.U.I. Ospedale Maggiore & University of Verona, Verona, Italy

**Keywords:** COVID-19, Cytokines, Inflammatory response, Mesenchymal stromal cells, ScRNA-seq

## Abstract

Coronavirus disease 2019 (COVID-19) may result in a life-threatening condition due to a hyperactive immune reaction to severe acute respiratory syndrome-coronavirus-2 infection, for which no effective treatment is available. Based on the potent immunomodulatory properties of mesenchymal stromal cells (MSCs), a growing number of trials are ongoing. This prompted us to carry out a thorough immunological study in a patient treated with umbilical cord-derived MSCs and admitted to the Intensive Care Unit for COVID-19-related pneumonia. The exploratory analyses were assessed on both peripheral blood and bronchoalveolar fluid lavage samples at baseline and after cellular infusion by means of single-cell RNA sequencing, flow cytometry, ELISA, and functional assays. Remarkably, a normalization of circulating T lymphocytes count paralleled by a reduction of inflammatory myeloid cells, and a decrease in serum levels of pro-inflammatory cytokines, mostly of interleukin-6 and tumor necrosis factor-α, were observed. In addition, a drop of plasma levels of those chemokines essential for neutrophil recruitment became evident that paralleled the decrease of lung-infiltrating inflammatory neutrophils. Finally, circulating monocytes and low-density gradient neutrophils acquired immunosuppressive function. This scenario was accompanied by an amelioration of respiratory, renal, inflammatory, and pro-thrombotic indexes. Our results provide the first immunological data possibly related to the use of umbilical cord-derived MSCs in severe COVID-19 context.

**Supplementary Information:**

The online version contains supplementary material available at 10.1186/s13287-021-02376-9.

## Introduction

In a variable proportion of cases, severe acute respiratory syndrome-coronavirus (SARS-CoV)-2 infection triggers a hyperacute inflammatory response that may ultimately lead to respiratory and multiorgan failure [1]. The immunological hallmarks comprise lymphopenia [2] and a flurry of active molecules which gives rise to the so-called “cytokine storm” [3], largely dominated by interleukin (IL)-6 and tumor necrosis factor (TNF)-α [4]. This causes disruption of both epithelial and endothelial barriers that magnifies inflammation and hampers gas exchange [5]. Despite there currently being a number of therapeutic approaches under investigation [6], none have substantially changed patients’ outcome. Following the results of early studies carried out in China showing the safety of applying mesenchymal stromal cells (MSCs) in patients with moderate to severe COVID-19-related pneumonia [7, 8], we offered this option to a patient admitted to the Intensive Care Unit for COVID-19 respiratory failure and unresponsive to current therapy. The rationale of this option lies on the evidence of a powerful and multifaceted action of MSCs on virtually all cell populations involved in inflammatory cascade [9]. Noteworthy, MSCs have also proven of benefit in experimental studies on acute respiratory distress syndrome [10], while the safety of this treatment option has been shown in a phase 2a trial carried out in this clinical setting [11]. Although these findings point to a potential role of MSCs even in COVID-19, no information is available on their immunological effects in this specific clinical setting. Therefore, we carried out an in-depth immune profiling at both bronchoalveolar and circulating levels to move the first step towards the understanding of their therapeutic effects in this dismal condition.

## Methods

### Mesenchymal stromal cell administration

**Fig. 1:**
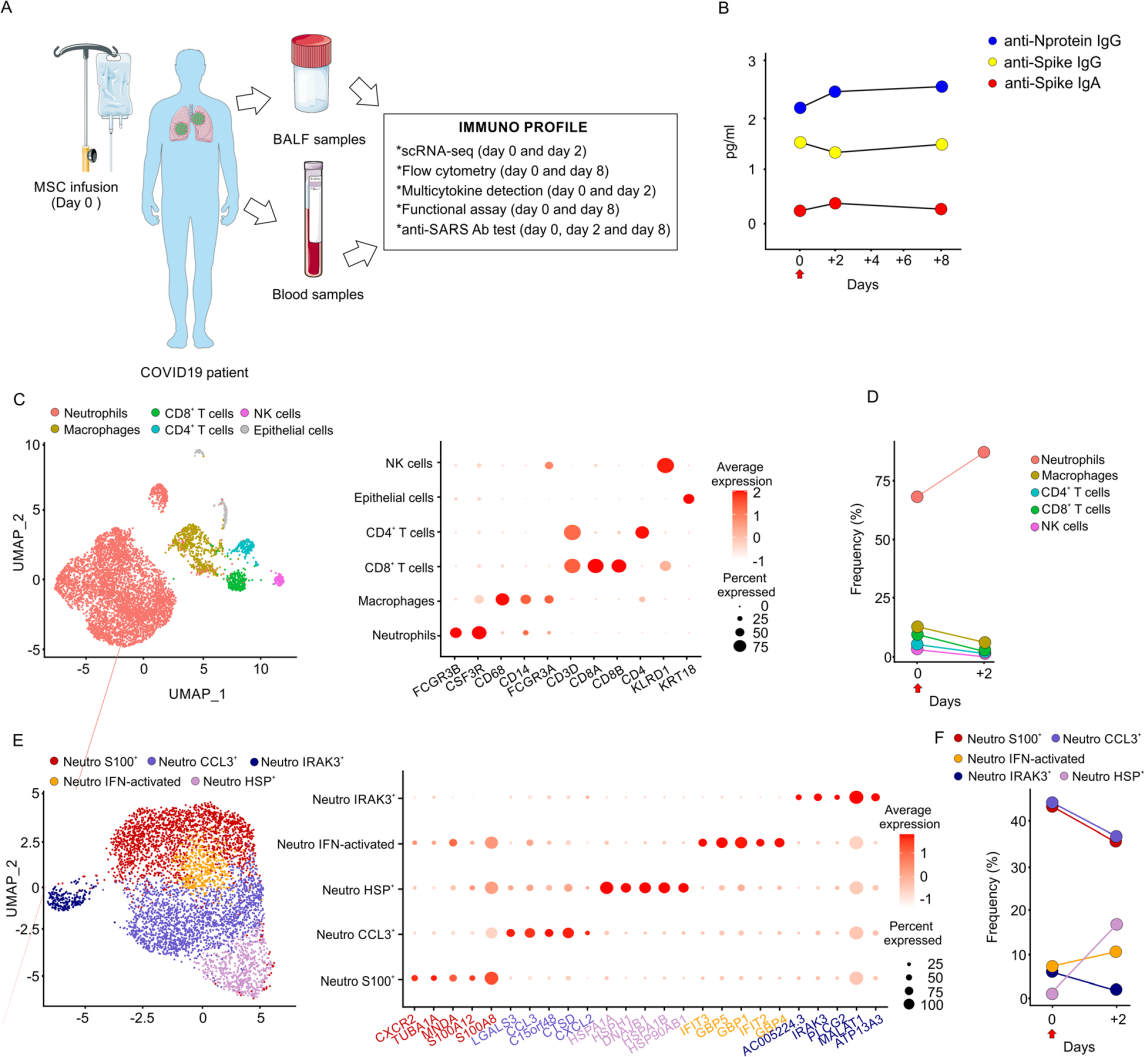
Study plan, serological and bronchoalveolar lavage fluid immune features. **a** Schematic representation of MSC administration and samples processing. **b** Plasma levels of anti-SARS-CoV-2 immunoglobulins at different time points (days 0, + 2, and + 8 after MSC infusion). **c** UMAP visualization of the six main cell types identified in the BALF by integrating the datasets of the days 0 and + 2 after MSC infusion. Dot plot graph shows the marker genes used to manually annotate each UMAP-associated cluster. Epithelial cells were discarded from further analyses. **d** Cell type frequencies in the days of observation. **e** The clustering of the BALF-derived neutrophils into five subgroups: S100^+^ neutrophils, CCL3^+^ neutrophils, IFN-activated neutrophils, IRAK3^+^ neutrophils, and HSP^+^ neutrophils. Dot plot graph shows the top five expressed genes by average log fold-change of each neutrophil sub-cluster. **f** The change in the proportion of each neutrophil sub-cluster in the days of observation. In **b**, **d**, and **f**, the red arrow indicates the time points in which MSC infusion was performed. For comments, see the text. BALF, bronchoalveolar lavage fluid; COVID, coronavirus disease; IFN, interferon; Ig, immunoglobulin; MSC, mesenchymal stromal cell; NK, natural killer; SARS-CoV, severe acute respiratory syndrome-coronavirus; scRNA-seq, single-cell RNA sequencing; UMAP, Uniform Manifold Approximation and Projection

The advanced cell therapy medicinal product was supplied by the Laboratory for Advanced Cellular Therapies (Vicenza, Italy) in two bags as frozen, sterile, apyrogenic umbilical cord-derived MSC suspension (see Supplementary material). Upon receiving, the bags were thawed in succession under sterile conditions, diluted 1:1 (50 ml final) in a solution consisting of 38% saline, 10% human albumin, and 12% anticoagulant citrate dextrose solution (Terumo; Rome, Italy), connected to an infusion set, delivered to the Intensive Care Unit and then infused intravenously in 30 min each (Fig. 1a). The final dosage was of 1.1 × 10^6^ cells/per kg body weight, in accordance with that used either in an early study on COVID-19 patients [7], or in further immune-mediated conditions, such as graft-versus-host disease [12, 13], and on personal experience [14, 15]. The patient was a 69-year-old Caucasian man who had been admitted to the Intensive Care Unit (A.O.U.I. Ospedale Maggiore; Verona, Italy) 2 weeks earlier for respiratory failure due to diagnosis of COVID-19 (see Supplementary material) pneumonia. The lack of amelioration upon a course of anti-viral agents (lopinavir-ritonavir) plus hydroxychloroquine and steroids, together with antiaggregant/coagulant therapy, vasoactive agents and nine rounds of prono-supination, and the failure at the screening assessment for further experimental therapies led us to offer him MSC infusion under the Hospital Exemption rule (art. 28 European Regulation 1394/2007), after approval by both the local Ethics Committee (no. 19464) and the Italian Drug Agency (no. 163516409) was obtained, and upon both the consent to participate and for publication were obtained by a relative since the patient was under pharmacological coma. The immunological analyses were performed on both bronchoalveolar lavage fluid (BALF) and peripheral blood samples before and after MSC infusion (Fig. 1a). Specifically, we decided to split the analyses using two different timeframes by considering that cytokines display a short life-span (estimated of hours), while circulating cell composition and myeloid cell function require a shift on myelopoiesis that need longer times (estimated of days). In parallel, a number of clinical and laboratory parameters were also assessed.

### Single-cell RNA sequencing

After isolation of immune cells from both BALF and peripheral blood samples (see Supplementary material) at baseline and after 2 days from MSC infusion, the single-cell RNA sequencing was carried out on 12,000 cells after reverse transcription, cDNA amplification, and library construction were performed (see Supplementary material). Sequencing, alignment, barcode processing, and unique molecular identifier quantification were then assessed after checking the sample quality. Following integration and normalization of collected data [16], the clustering analysis was built up and the Wilcoxon rank-sum test was used for computing the statistically significant difference (see Supplementary material). Analysis was performed at baseline and after 2 days from MSC infusion.

### Flow cytometry

Peripheral blood cell immunophenotyping was performed at baseline and 8 days after MSC infusion according to a standard no-wash procedure, using a PrepPlus™ 2 workstation (Beckman Coulter Inc.; Brea, CA, USA) by incubating the samples for 15 min at 20 °C with a panel of antibodies (all by Beckman Coulter Inc.) as listed in Supplementary material. Cytofluorimetric characterization of circulating immune cells was performed at baseline and after 8 days from MSC infusion.

### Detection of cytokines and serology

The following cytokines: IL-1β, IL-1RA, IL-2, IL-6, IL-8, IL-10, IL-15, interferon (IFN)-γ, TNF-α, vascular endothelial growth factor-A, and chemokines/ligands, CCL2, CCL3, CCL5, CXCL10, and CXCL12, were detected and quantified in the patient’s serum at baseline and after 2 days from MSC infusion by using the Human ProcartaPlex™ Panel 1 multiplex kit (Thermo Fisher Scientific; Waltham, MA, USA). Cytokine serum levels were detected at baseline and after 2 days from MSC infusion.

The ELISA assay to detect immunoglobulins (Ig) used fragment of the SARS-CoV2 spike glycoprotein (S-protein) as recently published [17].

### Functional assay

Upon Ficoll-Hypaque (GE Healthcare; Uppsala, Sweden) gradient centrifugation of peripheral blood samples harvested at baseline and 8 days after MSC infusion, monocytes and low-density gradient neutrophils (LDNs) were isolated by using CD14-microbeads (Miltenyi Biotec; Bergisch Gladbach, DE), and the sequential addition of CD66b-FITC antibody (BD Biosciences; NJ, USA) and microbeads anti-FITC (Miltenyi), respectively, following the manufacturers’ instructions. The purity of each fraction was evaluated at flow cytometry and a minimum cutoff of 95% was needed to perform the immunosuppressive assay [18]. A sample of 2 × 10^6^ cells of each cell type was seeded in 24-well plates for 12 h; afterwards, both the supernatants and cells were collected and cultured with CellTrace (Thermo Fisher Scientific; Waltham, MA, USA). Labeled cells were then stimulated with coated anti-CD3 (OKT-3) and soluble anti-CD28 for 4 days at 37 °C in 8% CO_2_. For the co-cultures, a 3:1 (target to effector) ratio was used. Cells were finally stained with anti-CD3-PE/Cy7 and CellTrace signal was analyzed with FlowJo software (Tree Star, Inc., Ashland). This analysis was performed at baseline and after 8 days from infusion.

## Results

### Clinical findings

Although SARS-CoV-2 N gene was detected in BALF samples at very high cycle threshold throughout the observational period, an improvement of the inflammatory, respiratory, thrombotic, and renal parameters was observed after 2 and 8 days after MSC infusion (Table 1). No adverse events occurred during the same time frame. In addition, neither the serum level of class A and G immunoglobulins specific for the receptor-binding domain of the SARS-CoV-2 spike protein nor that of class G antibodies recognizing the nucleocapsid phosphoprotein of the virus underwent modification (Fig. 1b).

**Table 1:** Clinical parameters

	Day 0	Day + 2	Day + 8
**SOFA score**	4	3	3
**Creatinine**	2.27	0.99	0.90
**HCO**_**3**_^**−**^	28.7	32.9	41.4
**D-Dimer**	3.83	2.68	1.32
**Prothrombin time**	1.18	1.09	0.96
**P/F**	168	136	114
**FiO**_**2**_	0.50	0.50	0.70
**pH**	7.31	7.34	7.38
**Base excess**	1.70	5.90	14.2
**White blood cells**	11.19	10.14	8.62
**Neutrophils**	9.29	8.28	6.59
**Lymphocytes**	1.01	1.11	1.21
**Monocytes**	0.37	0.44	0.40
**Basophils**	0.06	0.05	0.04
**Eosinophils**	0.46	0.26	0.38
**Red blood cells**	4.16	4.13	3.91
**Platelet**	365	327	287
**C-reactive protein**	201	139	61

*SOFA* Sequential Organ Failure Score, *P/F* oxygen partial pressure/oxygen inspiratory flow, *FiO*_*2*_ oxygen inspiratory flow

Normal values: creatinine, 0.49–1.19 mg/dL; HCO_3_^−^, 24–28 mmol/L; D-Dimer, < 0.25 mg/L; prothrombin time, 0.8–1.17 INR%; P/F, > 300 mmHg; FiO_2_, > 21%; pH, 7.35–7.45; base excess, − 2 to + 2 mmol/L; white blood count, 4.30–10 × 10^9^/L; neutrophils, 1.80–8 × 10^9^/L; lymphocytes, 1.20–4 × 10^9^/L; monocytes, 0.20–1 × 10^9^/L; basophils, < 0.20 × 10^9^/L; eosinophils, < 0.45 × 10^9^/L; red blood count, 4.00–5.20 × 10^12^/L; platelet, 150–450 × 10^9^/L; C-reactive protein, < 5 mg/L

Notably, at baseline, the patient was in respiratory acidosis (pH, 7.31; PCO_2_, 57 mmHg; BE, 1.70; HCO_3_^−^, 28.7 mmol/L), partially compensated by metabolic alkalosis on the second day (pH, 7.34; PCO_2_, 61 mmHg; BE, 5.9; HCO_3_^−^, 32.9 mmol/L). Then, the patient underwent continuous infusion of furosemide which led to a mixed clinical picture of metabolic alkalosis and respiratory acidosis, as exemplified by parameters of day 8 (pH, 7.38; PCO_2_, 70 mmHg; BE, 14.2; HCO3^−^, 41.4 mmol/L).

### Single-cell RNA sequencing of lung immune microenvironment

At total of 6362 high-quality single-cell transcriptomes for BALF cells were assessed and the overall population structure was visualized in two-dimensional space using Uniform Manifold Approximation and Projection (Fig. 1c). The unsupervised analysis identified six major clusters that were assigned to neutrophils (FCGR3B^+^, CSF3R^+^), macrophages (CD68^+^, FCGR3A^+^, CD14^+^), CD8^+^ T cells (CD3D^+^, CD8A^+^, CD8B^+^), CD4^+^ T cells (CD3D^+^, CD4^+^), natural killer (NK) cells (FCGR3A^+^, KLRD1^+^), and KRT18^+^ epithelial cells (Fig. 1d). In line with previously reported data on severe COVID-19 patients [19], BALF contained high proportions of neutrophils and macrophages with very limited number of T lymphocytes and NK cells (Fig. 1c). Notably, the frequency of lung-infiltrating immune cell populations did not substantially change during the observational time (Fig. 1d). However, a robust change became evident at a deeper analysis of neutrophils (Fig. 1e) since, according to the expression of typical markers, we identified two clusters of highly inflammatory neutrophils (S100^+^ neutro and CCL3^+^ neutro), one cluster of IFN-activated neutrophils (ISGs, GBPs) expressing CD274 (PD-L1) and one containing stressed/exhausted cells characterized by the production of heat shock proteins (HSP^+^ neutro), together with a small cluster of immature neutrophils (CD16^−^, CD10^−^) expressing IRAK3 (IRAK3^+^ neutro) (Fig. 1e). Interestingly, the frequency of highly inflammatory clusters along with the immature one underwent reduction after MSC infusion whereas a slight increase of the other two clusters occurred during the same interval (Fig. 1f).

### Single-cell RNA sequencing of circulating immune cells

**Fig. 2:**
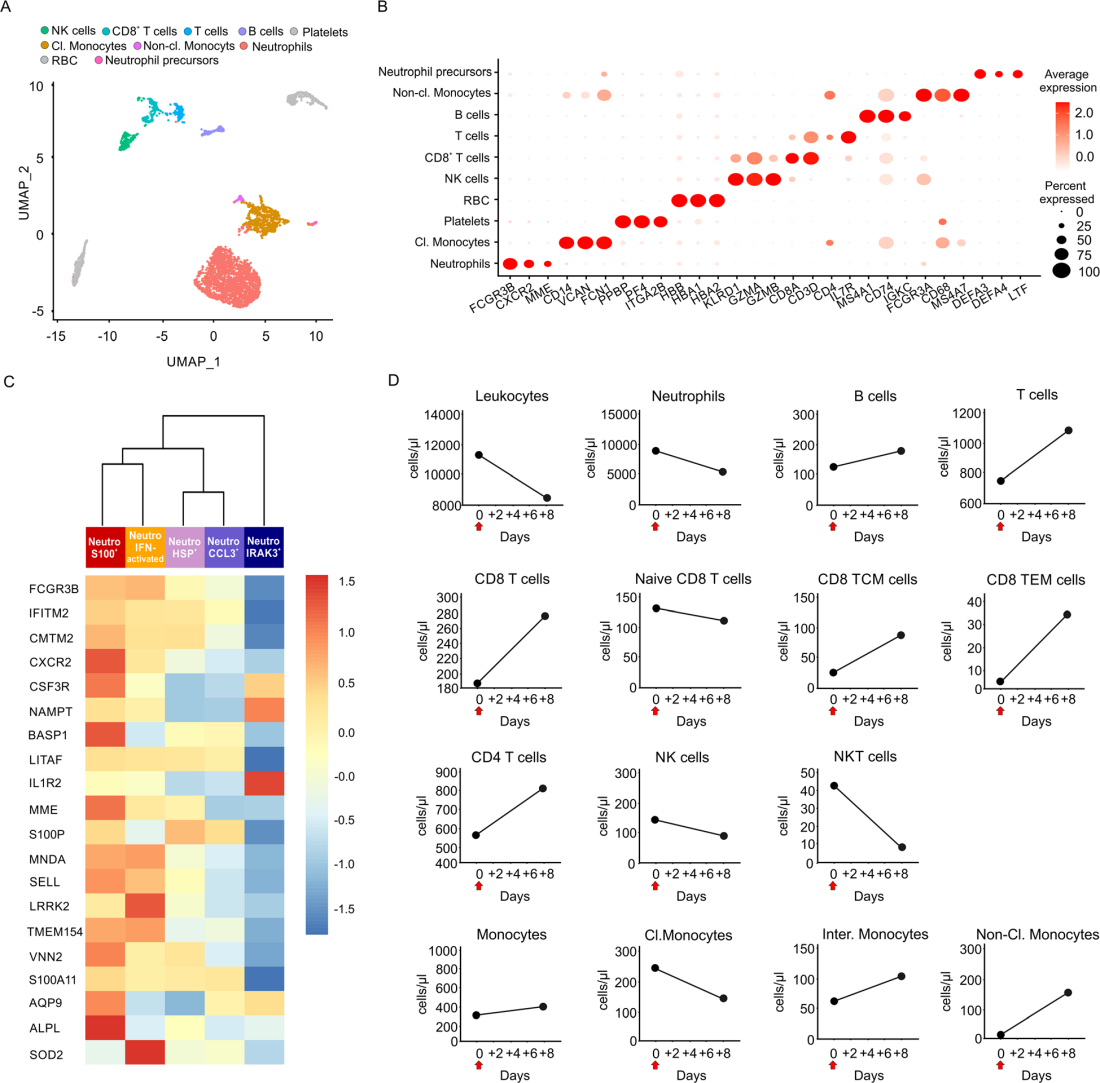
Circulating immune cell population characterization. **a** UMAP visualization of the 10 cell types identified by integrating the datasets of the days 0 and + 2 after MSC infusion. **b** Dot plot graph showing the top three marker genes for each cell population used to manually annotate each UMAP-associated cluster. **c** Top 20 marker genes by average log fold-change of the blood-derived neutrophil cluster and heatmap with their mean normalized expression in the bronchoalveolar lavage fluid-derived neutrophil sub-clusters. The heatmap is scaled for rows. **d** Peripheral blood samples were analyzed at day 0 and at day + 8 after MSC infusion by flow cytometry. Numbers of cells/μl were reported for leukocytes, neutrophils, B cells, T cells, CD8^+^ T cells, CD4^+^ T cells, naïve CD8^+^ T cells, central memory CD8^+^ T cells, effector memory CD8^+^ T cells, NK cells, NKT cells, monocytes, Cl. monocytes, intermediate monocytes and non-Cl. monocytes. In **b**, **d**, and **f**, the red arrow indicates the time points in which MSC infusion was performed. For comments, see the text. Cl., classic; IFN, interferon; Inter., intermediate; MSC, mesenchymal stromal cell; NK, natural killer; RBC, red blood cells; TEM, T-effector memory; UMAP, Uniform Manifold Approximation and Projection

A total of 3762 single-cell transcriptomes of peripheral blood cells were analyzed. The overall representation of each population showed 10 different clusters (Fig. 2a) and top three genes expressed in each cluster (Fig. 2b). Again, very few lymphoid cells were found in the following clusters: NK cells (FCGR3A^+^, KLRD1^+^, GZMA^+^, GZMB^+^), CD8^+^ T cells, T cells (CD4^+^, CD8^+^), and B cells (MS4A1^+^). The myeloid compartment was predominantly composed by classic monocytes (CD14^+^), inflammatory neutrophils (FCGR3B^+^), a small cluster of non-classic monocytes, and neutrophil precursors (CEACAM8^+^, ARG1^+^, MPO^+^). Interestingly, circulating neutrophils were mostly denoted by FCGR3B, CXCR2, and CSF3R resembling the BALF cluster of highly inflammatory neutrophils (S100^+^ neutro) (Fig. 2c) in line with previous reports [20, 21]. As shown in Fig. 2d, after MSC infusion, a contraction of the absolute number of circulating leukocytes occurred, mostly due to a decrease in neutrophils as well as NK and NKT cells, as evident at flow cytometry. By contrast, T cells increased as effect of expansion of both CD8^+^ and CD4^+^ subsets, mostly central memory CD8^+^ T cells (CD3^+^, CD8^+^, CD27^+^, CD45RA^−^) and effector memory CD8^+^ T cells (CD3^+^, CD8^+^, CD45RA^−^, CD57^+^), paralleled by a concurrent drop in naïve CD8^+^ T cells (CD3^+^, CD8^+^, CD27^+^, CD45RA^+^). Notably, a redistribution of monocyte subsets did occur since an increase of the absolute number of non-classic and intermediate monocytes and a decrease of classic ones were observed. By contrast, B-cell population was barely affected by MSC infusion.

**Fig. 3:**
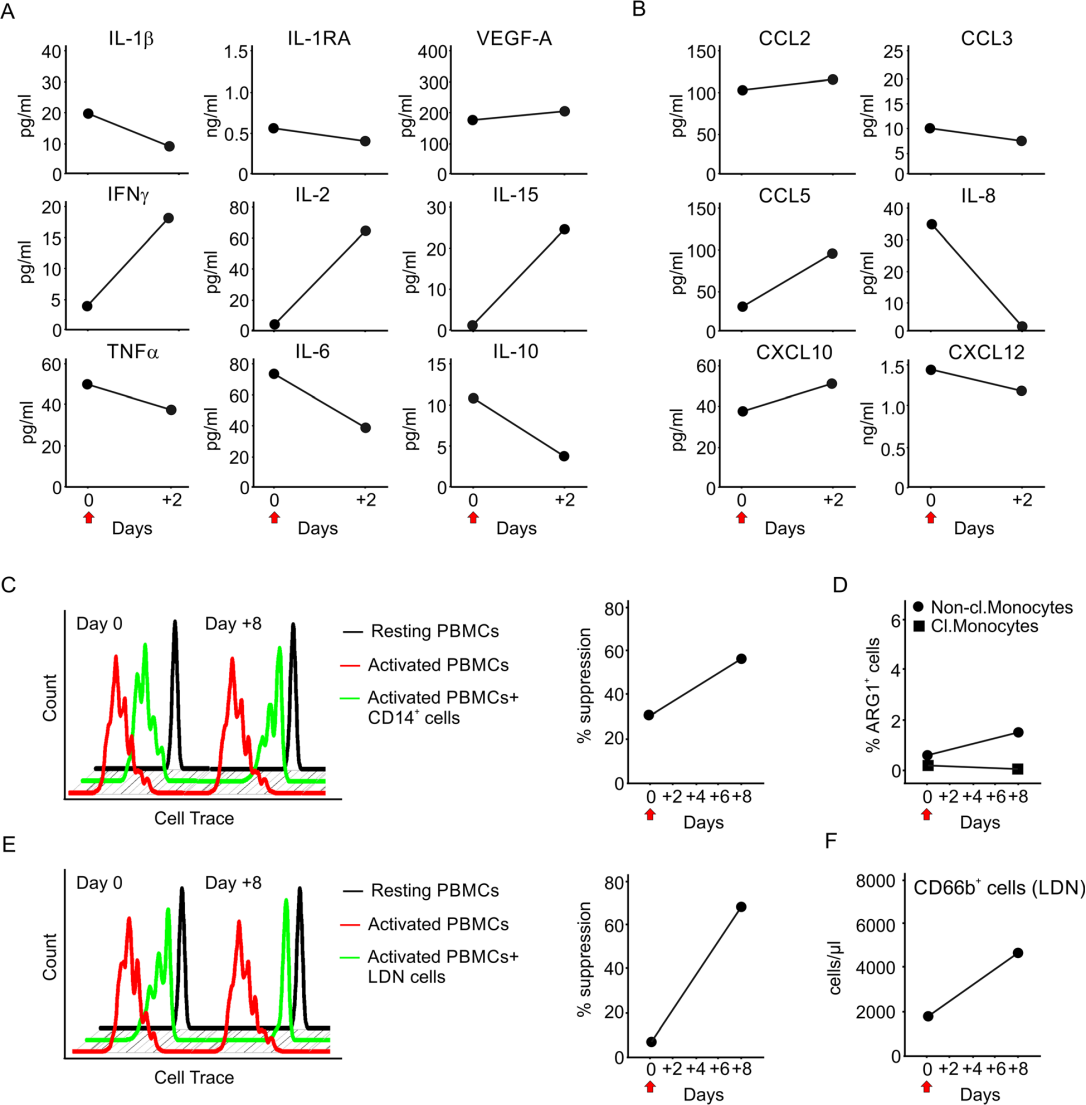
Plasma cytokine/chemokine profile, and functional assay. **a** Plasma levels of cytokines at days 0 and + 2 after MSC infusion. **b** Plasma levels of chemokines at days 0 and + 2 after MSC infusion. **c** The immune suppressive ability of monocytes was tested in functional assay on activated T cells and shown as percentage of suppression. **d** Arginase1^+^ cells within the monocytes subsets were evaluated by flow cytometry at different time points. **e** The immune suppressive ability of CD66b^+^ LDNs was tested in functional assay on activated T cells and shown as percentage of suppression. **f** LDNs were counted and reported as number of cells/μl. The red arrow indicates the time point in which MSC infusion was performed. For comments, see the text. CCL, C-C motif chemokine ligand; CXCL, C-X-C motif chemokine ligand; Cl., classic; IL, interleukin; IFN, interferon; IL-1RA, interleukin-1 receptor A; LDN, low-density gradient neutrophil; MSC, mesenchymal stromal cell; PBMC, peripheral blood mononuclear cell; TNF, tumor necrosis factor; VEGF, vascular endothelial growth factor

In parallel, a critical reduction of IL-1β, IL-1RA, IL-6, and TNF-α concentration was evident 2 days after MSC infusion, mimicking the effectiveness of other immunomodulatory agents, such as baricitinib [17], in dampening COVID-19-associated inflammation. Moreover, a decrease in IL-10 in combination with an opposite trend for IL-2, IL-15, and IFN-γ was detected during the same time frame, strengthening the idea that MSCs might fuel a favorable environment for T-cell proliferation (Fig. 3a), as indeed found (Fig. 2d). In addition, we identified increased levels of CCL2 (an essential chemokine for both monocyte and memory CD8^+^ T-cell migration and function), CCL5 (a potent chemoattractant for effector memory CD8^+^ T cells), and CXCL10 (an IFN-γ-inducible protein associated with type 1 T-cell response) [22] (Fig. 3b). By contrast, a drop on plasma levels of such chemokines (CCL3, IL-8, and CXCL12) essential for neutrophil recruitment [22] was clearly evident (Fig. 3b). These data might explain the decrease of lung-infiltrating inflammatory neutrophils (S100^+^ neutro and CCL3^+^ neutro) emerging from the single-cell RNA sequencing (Fig. 1f).

### Functional assay

Because the in vivo therapeutic effects of MSCs have been related to the ability of endogenous phagocytes to engulf apoptotic MSCs and activate an immunomodulatory program [23], we also performed an in vitro assay by using circulating monocytes (CD14^+^ cells) and LDNs harvested before and 8 days after MSC infusion. As shown in Fig. 3c, monocytes acquired the ability to suppress T-cells. This immunosuppressive feature seemed related to the increase of arginase1-expressing non-classic monocytes (Fig. 3d). Similarly, LDNs also acquired immunosuppressive properties (Fig. 3e), while their count gradually expanded (Fig. 3f).

## Discussion

Reversing of the “cytokine storm” appears a key strategy to counterattack COVID-19 pneumonia [1, 24]. MSCs have been widely explored as a new treatment option for a number of immune-mediated conditions, including acute steroid-refractory graft-versus-host disease, where a “cytokine storm” is responsible for the high mortality rate [25]. It is conceivable, therefore, that MSCs may also have positive effects on the “cytokine storm” observed in critically ill COVID-19 patients. Therefore, we decided to treat a patient under mechanical ventilation for COVID-19-related respiratory failure with umbilical cord-MSCs under the Hospital Exemption rule. It is recognized that upon intravenous administration, MSCs lodge in the pulmonary vascular bed, where the majority of them are cleared within 24–48 h [26], although there can be longer persistence in inflamed lungs [27]. This is why we chose to analyze the immunological parameters before and after 2 and 8 days after MSC infusion, on the basis of their estimated half-life. While trapped in the lungs, MSCs are able to release a wide array of bioactive molecules, including anti-inflammatory cytokines [28], antimicrobial peptides [29], angiogenic growth factors, and extracellular vesicles [30], that guarantee the phenotypic and functional shift of both epithelial and immune cells [31, 32]. Moreover, despite the fact that the effects of systemic MSC administration in animal models of influenza respiratory infections are under debate [33], systemic infusion of MSCs in a mouse model of lipopolysaccharide-induced ARDS significantly ameliorated alveolar injury and inflammation [34]. In line with this evidence, further studies showed that MSCs promoted the regeneration of alveolar epithelial type II cells, prevented the apoptosis of endothelial cells, and contributed to the enhanced repair of the alveolar epithelial barrier in the ARDS-injured lungs [35–37]. Although we cannot exclude that the modification observed upon MSC treatment was unrelated to cellular therapy, it is conceivable that the reduction of the levels of chemokines and inflammatory cytokines in serum samples (Fig. 3a, b) and the contraction of pro-inflammatory neutrophil subset at pulmonary system (Fig. 1e, f) mirrored by an improvement of gas exchange and clinical parameters were the direct or indirect effects of MSC action. Moreover, our data suggest that MSCs administration was temporally associated with the development of favorable microenvironment for T-cell proliferation, while restraining the accumulation of pro-inflammatory neutrophils that could have actively fueled inflammation during the disease progression. In addition, monocytes underwent re-programming towards the intermediate/non-classical phenotype, a subset with more immunomodulatory function [38] and a higher propensity to become dendritic cells with a strong capacity to induce T-cell proliferation [39, 40]. This is an important point since there is growing evidence showing the relevant role played by monocytes in MSC-driven immunomodulation [41]. Further, our in vitro assay showed the ability of both monocytes and LDNs to acquire immunosuppressive abilities upon MSC treatment. In this regard, it should be emphasized that an expansion of arginase1-expressing CD14+ cells was found, a monocyte subset that we recently identified as immunosuppressive elements in pancreatic cancer patients [42].

However, an important question that remains to be solved is whether protective MSC effects are directly against viral infection, perhaps by stimulating specific anti-viral T-cell action, or whether they are due to overall anti-inflammatory effects. Following the persistence of detectable levels of the N gene in the patient’s BALF samples, it is likely that MSCs favor the development of an anti-inflammatory and protective environment able to dampen the “cytokine storm” while contributing to the restoration of alveolar-capillary barrier, instead of having a direct anti-viral effect.

Although the data presented here were obtained from a single patient, the completeness and depth of the immunological analyses carried out reinforces the idea of an altered myeloid compartment in the specific setting of COVID-19 severe pneumonia [21]. Moreover, the parameters identified may be of meaningful clinical utility as a potential platform to identify key biomarkers to design consistent and informative clinical trials on the use of MSC therapy in this dismal condition.

## Supplementary Information

**Additional file 1: Supplementary methods.**

## Data Availability

The data that support the findings of this study are available from the corresponding author upon reasonable request.

## Abbreviations

BALF: Bronchoalveolar lavage fluid
CCL: C-C motif chemokine ligand
CXCL: C-X-C motif chemokine ligand
COVID: Coronavirus disease
IFN: Interferon
IL: Interleukin
LDNs: Low-density neutrophils
MSC: Mesenchymal stromal cell
NK: Natural killer
SARS-CoV: Severe acute respiratory syndrome-coronavirus
TNF: Tumor necrosis factor
