# Supplementary material for: The immune modulatory effects of umbilical cord-derived mesenchymal stromal cells in severe COVID-19 pneumonia
Source: Stem Cell Res Ther. 2021 Jun 2;12:316. doi: 10.1186/s13287-021-02376-9 (PMC8170427; doi:10.1186/s13287-021-02376-9)
Supplement: Supplementary file 1 — Additional file 1: Supplementary methods. [file 13287_2021_2376_MOESM1_ESM.docx]

**SUPPLEMENTARY MATERIAL**

**Mesenchymal stromal cell manufacturing**

Clinical grade mesenchymal stromal cell (MSC) batches were produced at the Laboratory for Advanced Cellular Therapies (Vicenza, Italy) according to Good Manufacturing Practice (authorization code: aM-49/2019, aM-49bis/2019). Briefly, after mother’s informed consent (Ethics Committee act no. 16/18 of 13 February 2018) was obtained, and screening for hepatatis B and C viruses, human immunodeficiency virus, and *Treponema pallidum* resulted negative, the umbilical cord was collected after caesarean section and immediately submerged in a decontamination solution containing a cocktail of four antibiotics (BASE 128, Alchimia; Padua, Italy). Then, in a clean room, the umbilical cord was minced into smaller pieces and seeded on conventional T-flasks in culture medium containing human-derived platelet lysate (produced by the blood bank of Meyer Hospital; Florence, Italy) at 37°C in 5% CO_2_. After seven days, tissue fragments were removed and medium was substituted. After further 13 days, adherent cells were detached by using TrypLe Select (Thermo Fischer; Waltham, MA, USA) and re-seeded in complete medium in HYPERFlask Cell Culture Vessels (Corning; NY, USA). On day 26^th^, cells were harvested, counted and collected in CryoMACS freezing bags (Miltenyi Biotec; Bergisch Gladbach, Germany). Cells were finally resuspended at a concentration of 1 or 2x10^6^ cells/ml in 25 ml of freezing solution (80% v:v saline, 2% v:v human albumin; 10% v:v dimethyl sulfoxide) and frozen to -140°C in a cryogenic freezer (Feezal, Air Liquide; Paris, France) at a cooling rate of -1°C/min to -40°C, followed by a cooling rate of -5°C/min to -140°C, and then stored in liquid nitrogen vapour phase. MSC bags were released upon sterility, mycoplasma, endotoxins, cell count, phenotype, karyotype, and cell viability were assessed following the European Pharmacopeia methods. After validation of the supply chain **[1]**, the cellular preparation was used to be infused in a patient suffering from respiratory failure due to COVID-19 pneumonia.

**Severe acute respiratory syndrome-Coronavirus-2 detection**

Nucleic acids were extracted from bronchoalveolar lavage fluid (BALF) samples by Seegene Nimbus instrument (Seegene; Seoul, South Korea) and the target amplification was performed by Allplex 2019-nCoV assay kit (Seegene), a multiplex polymerase chain reaction procedure for the simultaneous detection of E, N and RdRP severe acute respiratory syndrome coronavirus-2 genes, following manufacturer’s instructions.

**Bronchoalveolar lavage fluid and peripheral blood cell isolation**

For each time point, approximately 20 ml of bronchoalveolar lavage fluid (BALF) was collected and processed within two hours in a BSL-3 laboratory. The sample was first filtered two times using a 100-µm nylon cell strainer to remove clumps and debris. The supernatant was then washed with PBS 1× and centrifuged. Peripheral blood samples were collected in EDTA-coated tubes and washed once with PBS 1×. In both sample types red blood cells were lysed for three minutes at room temperature with 0.2% NaCl solution and the reaction was blocked by adding 1.2% NaCl solution. The cells were finally washed with PBS 1×, re-suspended in RPMI 1640 medium supplemented with 5% bovine serum albumin and counted. Cell viability was determined by Trypan blue exclusion, and viability higher than 95% was considered appropriate. For single cell analysis, cells were re-suspended at a concentration of 1×10^6^ /ml.

**Single-cell RNA sequencing**

For each BALF and peripheral blood sample, 12,000 cells were loaded in the ChromiumTM Controller in order to partitioning single cells into Gel Bead-In-Emulsions (10x Genomics; Pleasanton, CA, USA). Single Cell 3’ reagent kit v3.1 was used for reverse transcription, cDNA amplification and library construction of the gene expression libraries were performed following the manufacturer’s instructions. GenePro Thermal Cycler was applied for amplification and incubation steps (Bioer Technology Co., Ltd.; Hangzhou, Zhejiang, China). Libraries were quantified by quantitative polymerase chain reaction (KAPA Biosystems Library Quantification Kit for Illumina platforms) and quality was checked using Bioanalyzer with High Sensitivity DNA kit (Agilent; Santa Clara, CA, USA). Sequencing was performed in paired-end mode using NextSeq 500 sequencer (Illumina; San Diego, CA, USA). The bcl2fastq software (v2.20.0.422) provided by Illumina (<https://support.illumina.com/sequencing/sequencing_software/bcl2fastq-conversion-software.html>) was used for sample demultiplexing and to obtain the raw FASTQ files. Alignment, barcode processing and unique molecular identifier (UMI) quantification was performed through the Cell Ranger software (v.3.1.0) (<http://software.10xgenomics.com/single-cell/overview/welcome>) using the human GRCh38 as reference genome. In particular, the ‘cellranger count’ pipeline was executed for each BALF and peripheral blood sample specifying the parameter ‘--expect-cells=6,000’. All the subsequent analyses were performed using the Seurat v.3 R package. BALF and peripheral blood samples were individually preprocessed prior to integration. For both kind of samples, only the cells with mitochondrial gene percentage <20% were considered for further analyses. In addition, the following filtering criteria were used to maintain high quality cells: gene number >150 or >60 and UMI count <35.000 or <30.000 for BALF and blood samples, respectively. BALF and peripheral blood results were then integrated using the Seurat method [2] in order to make the cells comparable and to avoid technical bias due to the different sequencing runs. The integration was performed using the first 30 dimensions of canonical correlation analysis. The integrated barcode matrices were normalized using the Seurat function ‘NormalizeData’ with default parameters. Next, the top 2,000 variable genes were identified using the function ‘FindVariableFeatures’. Prior to visualize the cells, matrices were scaled regressing out for the number of genes, UMI and mitochondrial gene percentage in order to remove unwanted variation. Then, a Uniform Manifold Approximation and Projection (UMAP) was executed on the top 21 principal components and ‘min.dist’ parameter of 0.3 for BALF and top 17 principal components and ‘min.dist’ parameter of 0.2 for pheripheral blood. The same principal components were used to construct a shared nearest neighbor graph through the function ‘FindNeighbors’ that was used for clustering analysis through the function ‘FindClusters’, after setting the resolution parameter to 0.2. Clustering analysis resulted in 9 clusters for BALF and 10 clusters for peripheral blood corresponding respectively to 6 and 10 major cell types. For BALF samples, neutrophils isolation and re-clusterization at a resolution of 0.3 gave rise to five sub-clusters. Gene markers for each cluster were obtained using the Wilcoxon rank-sum test implemented in the function ‘FindAllMarkers’ keeping only up-regulated genes with an adjusted p-value < 0.05.

**Flow cytometry analysis**

List of the antibodies used for the immunophenotyping of circulating immune cells (all by Beckman Coulter Inc.: Brea, CA, USA):

● FITC-conjugated CD57 (clone NC1), PE-conjugated CD45RA (ALB11), ECD-conjugated CD8 (SFCI21Thy2D3), PC5.5-conjugated CD56 (N901), PC7-conjugated CD4 (SFCI12T4D11), APC-conjugated CD27 (1A4CD27), APC A700-conjugated CD45 (J33), APC A750-conjugated CD3 (UCHT1), PB-conjugated CD16 (3G8), KR OR-conjugated CD19 (J3-119).

● FITC-conjugated CD57 (clone NC1), PE-conjugated HLA-DR (Immu-357), ECD-conjugated CD8 (SFCI21Thy2D3), PC5.5-conjugated CD38 (LS198-4-3), PC7-conjugated CD4 (clone SFCI12T4D11), APC-conjugated CD14 (RMO52), APC A700-conjugated CD45 (J33), APC A750-conjugated CD3 (UCHT1), PB-conjugated CD16 (3G8), KR OR-conjugated CD19 (J3-119).

● PE-conjugated IgD (IA6-2), ECD-conjugated CD3 (UCHT1), PC5.5-conjugated CD27 (1A4CD27), PC7-conjugated CD20 (clone B9E9), APC-conjugated IgM (SA-DA4), APC A700-conjugated CD45 (J33), APC A750-conjugated CD38 (LS198-4-3), PB-conjugated CD21 (BL13), KR OR-conjugated CD19 (J3-119).

Red blood cell lysis and cell fixation were performed using TQ-Prep workstation and ImmunoPrep reagent system (Beckman Coulter Inc.). Data acquisition and analysis was performed using Navios and Navios software, respectively (Beckman Coulter Inc.).

The amount of monocyte subsets (defined as classical: CD14^high^ CD16^low/dim^; intermediate: CD14^int^ CD16^+^; non classical: CD14^low/dim^CD16^high^) in the peripheral blood was assessed using FcR Blocking reagent followed by the addition of: anti-human PE-conjugated CD56 (NCAM16.2), FITC-conjugated-CD16 (3G8), PerCP-Cy5.5-conjugated CD3 (UCHT1), PE.Cy7-conjugated HLA-DR (L243), APC.H7-conjugated CD14 (MφP9), Brilliant Violet 421™-conjugated PD-L1 (MIH1) antibodies and Aqua LIVE/DEAD dye (ThermoFisher Scientific; Waltham, MA, USA). Red blood cells were lysed using Cal-Lyse™ Lysing Solution (ThermoFisher Scientific) according with the manufacturer’s instructions. Samples were acquired with FACS Canto II (Beckton Dickinson; Franklin Lakes, NJ, USA) and analyzed with FlowJo software (Tree Star, Inc.; Ashland, OR, USA).

**REFERENCES**

1. Astori G, et al. Logistics of an advanced therapy medicinal product during COVID-19 pandemic in Italy: successful delivery of mesenchymal stromal cells in dry ice. J Translat Med. 2020;18:251. <https://doi.org/10.1186/s12967-020-02625-0>.
2. Stuart T, et al. Comprehensive Integration of Single-Cell Data. Cell. 2019;177(7):1888-902. doi: [10.1016/j.cell.2019.05.031](https://doi.org/10.1016/j.cell.2019.05.031).
